# Trypsin inhibitor LH011 inhibited DSS-induced mice colitis *via* alleviating inflammation and oxidative stress

**DOI:** 10.3389/fphar.2022.986510

**Published:** 2022-09-27

**Authors:** Zhenmao Jia, Panxia Wang, Yuansheng Xu, Guodong Feng, Quan Wang, Xiangjun He, Yan Song, Peiqing Liu, Jianwen Chen

**Affiliations:** ^1^ Laboratory of Pharmacology and Toxicology, School of Pharmaceutical Sciences, Sun Yat-sen University, Guangzhou, China; ^2^ School of Pharmaceutical Sciences, Guangzhou Medical University, Guangzhou, China; ^3^ Guangzhou Link Health Group, Guangzhou, China; ^4^ Department of National and Local United Engineering Lab of Druggability and New Drugs Evaluation, School of Pharmaceutical Sciences, Sun Yat-Sen University, Guangzhou, China; ^5^ Guangdong Provincial Key Laboratory of New Drug Design and Evaluation, School of Pharmaceutical Sciences, Sun Yat-Sen University, Guangzhou, China; ^6^ Guangdong Provincial Key Laboratory of Chiral Molecule and Drug Discovery, Guangzhou, China

**Keywords:** UC, LH011, inflammation, oxidative stress, NF-κB, Nrf2

## Abstract

**Background:** Ulcerative colitis (UC) is one type of inflammatory bowel disease, characterized by inflammation with infiltration and activation of macrophages in colonic tissue. LH011 is a trypsin inhibitor with potential anti-inflammatory effect.

**Purpose:** Here, we aim to assay the effects of LH011 on UC and further investigate the potential mechanisms *in vitro* and *in vivo.*

**Methods:** Dextran sulfate sodium (DSS, 3.5%, w/v) was used to induce UC, and lipopolysaccharide (LPS) was used to induce inflammation in RAW 264.7 cells. LH011 was administrated to mice *in vivo* or to RAW 264.7 cells *in vitro* at different concentrations. The cytokines (IL-1β, IL-6, and TNF-α) and the changes of NF-κB and Nrf2 pathways were detected.

**Results:** The results showed that LH011 improved DSS-induced mice colitis, including loss of weight, disease activity index (DAI), and colonic pathological damage. In addition, LH011 inhibited the expressions of IL-1β, IL-6, and TNF-α and strengthened the anti-oxidative capacity. Mechanically, LH011 downregulated the nuclear localization of NF-κB p65 and upregulated the protein expression of Nrf2.

**Conclusion:** These results demonstrated that LH011 alleviated inflammation and oxidative stress during UC by inhibiting TLR4/NF-κB and activating Nrf2/Keap1/HO-1 signaling pathways.

## Introduction

Ulcerative colitis (UC) is a common inflammatory bowel disease (IBD), which is an idiopathic inflammatory disease of the gastrointestinal tract, and it is characterized by diarrhea, abdominal pain, and bloody stool, or even weight loss and vomiting in the severe cases (Goodhand et al., 2012; Chaithanya et al., 2018). The incidence of UC is increasing with younger trend and persistent colitis might develop into colorectal cancer. The main incidence areas of UC always appear in the zone of the rectum and part of colon, with severe inflammatory responses and tissue damage (Ng et al., 2013). Generally, during the development of UC, infiltration and activation of macrophages in the colonic tissue play a key role in the excessive secretion of inflammatory factors and lead to severe colonic tissue damage (Yi et al., 2019). Although mesalazine, glucocorticoid, immunosuppressive drugs, or TNF-α monoclonal antibody could partially improve the symptoms of UC, the diverse side effects and wide patient tolerance limit the administration of these drugs (Xiao et al., 2019). Therefore, it is urgent to explore innovative therapeutic regimen and potential drugs for UC.

Acting as the main player in the first-line defense, macrophage is the key mediator of innate immunity and the pivotal regulator of intestinal microenvironment homeostasis (Muller et al., 2020). UC is closely related to immune dysfunction. During the development of UC, macrophages are recruited to the pathologic sites and polarized into M1 type macrophages, which prefers to aggregate disease progression by releasing pro-inflammatory cytokines including tumor necrosis factor-α (TNF-α) and interleukins (ILs) (Moghaddam et al., 2018). Many signaling pathways participated in adjusting the production of inflammatory cytokines and inflammatory mediators, such as NF-κB and Nrf2/Keap1/HO-1 (Wang et al., 2019; Chen et al., 2021). Moreover, strategies targeting macrophage phenotypes transition or regulating the metabolism functions alleviated the progression of UC, such as regulating macrophages polarization (Wu et al., 2021). Therefore, macrophage-based therapy during UC is a promising strategy.

WX-UK1 is a synthetic serine protease inhibitor with amidinophenylalanine-type, which inhibits urokinase-type plasminogen (uPA) activity. WX-UK1 has been considered as a potential agent for the treatment of solid tumors, such as breast cancer (Setyono-Han et al., 2005). WX-UK1 is also known as a trypsin inhibitor (International Publication Number was WO 2021/181157 A1). Many evidence have strongly indicated that abnormal elevation of trypsin is closely related to UC. A marked increase in trypsin-like activity was detected in colonic lavage and colonic tissue of animal models of UC (Zhou et al., 2017). Compared to activity detected in healthy individuals, data collected from tissue biopsies of patients with UC showed increased serine protease activity similar to trypsin-like enzyme (Dabek et al., 2009). Previous studies have demonstrated that antitrypsin therapy may become a new therapeutic strategy in human UC (Yoshida and Yoshikawa, 2008). However, there are few studies in the treatment of UC with trypsin inhibitors. Therefore, WX-UK1 has potential and novelty application in the treatment of trypsin-driven inflammatory digestive diseases, such as ulcerative colitis. Generally, WX-UK1 is administrated intravenously, which makes it inconvenient to patient. Therefore, the pro-drug LH011 was developed for oral delivery. LH011, also known as WX-671, is absorbed and then reductively converted into pharmacologically active WX-UK1 (Meyer et al., 2008).

In this study, the effects of LH011 in UC were assayed by the DSS-induced acute colitis *in vivo* model and LPS-induced RAW 264.7 cells *in vitro* inflammation model. Furthermore, NF-κB and Nrf2 signaling pathways were assayed. This study showed that LH011 effectively protected colitis by its anti-inflammatory and anti-oxidative stress effects. Mechanically, LH011 effectively inhibited the TLR4/NF-κB signaling pathway and activated the Nrf2/Keap1/HO-1 signaling pathway. Therefore, this study demonstrated that LH011 might be a novel compound to protect against ulcerative colitis.

## Materials and methods

### Reagents

LH011 (N-alpha-(2,4,6-triisopropylphenylsulfonyl)-3-hydroxyamidino-(l)-phenylalanine-4-ethoxycarbonylpiperazide) was obtained from Guangzhou Link Health Pharma Co., Ltd. (Guangzhou, China). Dextran sulfate sodium (DSS, MW: 36000–50000) was obtained from Meilunbio (Dalian, China). LPS (from *Escherichia coli* O55: B5) and sulfasalazine (SASP) were obtained from Sigma-Aldrich (St. Louis, MO, United States). The enzyme-linked immunosorbent assay (ELISA) kit for mouse TNF-α, IL-6, and IL-1β were purchased from Multisciences (Hangzhou, China). Malondialdehyde (MDA) (catalog: S0131) assay kit, superoxide dismutase (SOD) assay kit with WST-8 (catalog: S0101), and dual-luciferase assay system (catalog: RG027) were purchased from Beyotime Biotechnology (Shanghai, China). MPO (catalog: E-BC-K074) assay kit was purchased from Elabscience Biotechnology (Wuhan, China). The occult blood (OB) assay kit (catalog: BA 2020B) with pyramidon was purchased from BASO (Zhuhai, China). The primary antibodies against COX2 (catalog: 12282), iNOS (catalog: 13120), NF-κB p65 (catalog: ab16502), phospho-IκBα (catalog: 2859), IκBα (catalog: 4814), α-tubulin (catalog: 11224-1-AP), and lamin-B (catalog: ab13374) were purchased from Cell Signaling Technology (Boston, MA, United States). Nrf2 (catalog: 16396-1-Ap), Keap1 (catalog: 10503-2-AP), and HO-1 (catalog: 10701-1-Ap) antibodies were purchased from Proteintech Group (Chicago, IL, United States). The antibody against TLR4 (catalog: sc-293072) was purchased from Santa Cruz Biotechnology (Santa Cruz, CA, United States). The secondary antibodies conjugated with HRP, Alexa Fluor-488, and Alexa Fluor-594 goat antimouse IgG were obtained from Thermo Fisher Scientific, Inc. (Rockford, United States).

### Model and treatment of animals

For the sex-specific differences of colitis, in DSS-induced colitis, male mice are more sensitive than female mice (Wirtz et al., 2017). Therefore, male C57BL/J mice (6–8 weeks, 18–22 g) were purchased from Guangdong Yaokang Biotechnology Co., Ltd (SYXK 2021-0112, Guangdong, China). All mice were fed under SPF conditions with appropriate temperature (22 ± 2°C), humidity (60 ± 10%), and light and dark cycle (12 h). The *in vivo* experimental procedures were approved by the rules of animal ethics of Sun Yat-sen University and animal ethics code number was L102042021120L. Mice were randomly subdivided into six groups after adaptively fed for 7 days: control group (sterile water, n = 9), model group (3.5% DSS, n = 9), the low dosage group of LH011 (5 kg/mg, n = 9), the medium dosage group of LH011 (10 kg/mg, n = 9), the high dosage group of LH011 (20 kg/mg, n = 9), and the positive drug group of sulfasalazine (200 mg/kg, n = 9). To induce the colitis model, DSS (3.5% w/v) was dissolved in the sterile water and freely drank by mice for 7 days. Each mouse freely drank about 6–8 ml every day. LH011 is a non-hygroscopic, white powder which is slightly soluble in water. Ethanol as an organic solvent can increase the solubility of insoluble drugs. According to the Handbook of Pharmaceutical, the recommended concentration of ethanol for intraperitoneal injection is 5–10%. Therefore, LH011 was dissolved in normal saline with 1.5% ethanol at different concentrations (5, 10, 20 mg/kg) respectively and administrated to mice by intraperitoneal injection. SASP (200 mg/kg, p. o.) was dissolved in 0.5% carboxymethyl cellulose (CMC-NA) as the positive control. Mice in the control group were given sterile water and vehicle solvent. Weight changes, stool consistency, and bleeding of mice were recorded every day. After 7 days, all mice were anesthetized by inhaling 2% isoflurane for 2–3 min. After complete anesthesia, 1–2 ml blood was collected from the mice orbit, and then the mice were euthanized by cervical dislocation.

### Calculation of DAI

As described in previous studies (Funakoshi et al., 2012), DAI consisted of three parts: body weight loss(A), stool consistency (B), and stool bleeding (C). The changes of daily DAI scores were calculated as (A + B + C)/3. For body weight loss, scores 0, 1, 2, 3, and 4 were correspond to ≤1, 1–5, 5–10, 10–20, and >20%. As for stool consistency, 0 for normal, 1 for soft stools, 2 for loose stools, 3 for muddy stools, and 4 for diarrhea. About stool bleeding, negative, mild hemoccult, obvious hemoccult, and gross bleeding were scored for 0, 2, 3, and 4.

### Histological analysis

After the mice were sacrificed, their colons were separated, photographed, and measured. A portion of colonic tissue was fixed by 10% formalin and then paraffin embedded. Sections (4 μm thick slice) of colon were used for hematoxylin and eosin (H&E) staining. Remaining colonic tissues were frozen at -80°C for further analysis. Histopathological scores were calculated, according to percentage of tissue damage (0 = no tissue damage, 1 = 1–25%, 2 = 26–50%, 3 = 51–75%, and 4 = 76–100%), extent of crypt damage (1 = 1/3 damaged, 2 = 2/3 damaged, 3 = only surface epithelium intact, and 4 = entire crypt and epithelium absent), extent of tissue damage (1 = mucosa, 2 = mucosa and submucosa, and 3 = above submucosa), and the degree of inflammation (1 = mild, 2 = moderate, and 3 = severe) (Dieleman et al., 1998).

### MPO, MDA, and SOD measurement

The level of MPO was measured by the MPO commercial assay kit (Elabscience Biotechnology, Wuhan, China). Colonic tissues were homogenized immediately after weighing, and then centrifuged at 10,000 g for 10 min at 4°C. Subsequently, the absorbance was detected at 460 nm by using an ultraviolet visible spectrophotometer (Thermo Fisher Scientific, Vantaa, Finland). The MDA and SOD were measured by using assay kits (Beyotime, Shanghai, China), according to the manufacturer’s instructions.

### Cell culture and treatment

The RAW 264.7 cells (Cell bank of the Chinese Academy of Science, Shanghai, China) were cultured in DMEM medium with 10% FBS containing 100 U/mL streptomycin and 100 U/mL penicillin at 37°C incubator with 5% CO_2_. The cells were pre-treated with different concentrations of LH011 (2.5, 5, and 10 µM), and then co-stimulated with LPS (1 μg/ml) for the indicated time.

### Nucleic and cytoplasmic protein extraction

The RAW 264.7 cells (1×10^4^ cells/mL) were incubated with LH011 at different concentrations (2.5, 5, and 10 μM) for 24 h and then co-treated with LPS (1 μg/ml) for the last 1 h. The proteins of nucleus and cytoplasm were extracted by the commercial extraction kit (Active Motif, United States), according to the manufacturer’s instructions, and then conducted Western blot analysis after the concentrations of protein have defined quantitatively.

### Transfection of siRNA

The RAW 264.7 cells (1×10^4^ cells/mL) were plated in 35 mm plates for 24 h. The siRNA was transfected by using Lipofectamine 2000 reagent (Invitrogen, Carlsbad, CA, United States) protocol. Small interfering RNA (siRNA) against mouse Nrf2 and negative control (NC) siRNA were purchased from GenePharma (GenePharma, Shanghai, China) and the sequences of Nrf2-siRNA were: sense: 5′-CCG​AAU​UAC​AGU​GUC​UUA​ATT-3′, antisense: 5′-UUA​AGA​CAC​UGU​AAU​UCG​GTT-3’. After 4–6 h of incubation, the transfected medium was changed to fresh culture medium for another 48 h, and the cells were harvested for the next step.

### Real-time quantitative PCR (RT-qPCR) analysis

Total RNA of RAW 264.7 cells was extracted by RNAiso Plus (TaKaRa, CA, United States), and RT-qPCR was performed according to the manufacturer’s instructions. The concentration of RNA was tested by Nanodrop 2000 (Thermo Fisher Scientific, United States), and 1 μg of total RNA was reverse transcribed into cDNA by the RevertAid reverse transcriptase kit (Thermo Fisher Scientific, United States). The specific primers of genes were provided by Sangon Biotech Co., Ltd. (Shanghai, China) (Supplementary Table S1), while β-actin acted as a home keeper gene. The relative gene expression was normalized to β-actin expression.

### Western blotting analysis

The cells and the frozen colonic tissues were washed with PBS buffer (pH 7.4), and then lysed with RIPA buffer contained with protease and phosphatase inhibitors. The concentration of protein was determined by the BCA assay kit after centrifuge at 12,000 rpm at 4°C for 15 min. Equal amounts of protein were added in 10–12% SDS–PAGE gels to separate, and then transferred to PVDF membranes. Membranes were then blocked with 5% bull serum albumin (BSA) for 1 h at room temperature. Next, membranes were incubated with the primary antibodies overnight at 4°C. The membranes were incubated with the secondary antibodies at room temperature for 1 h after washing with TBST three times for 10 min. The blots were detected after incubating with an enhanced chemiluminescence (ECL) agent (Tanon, Shanghai, China). Densitometry of blots were analyzed by using ImageJ software.

### Statistical analysis

All experimental data were expressed as mean ± SEM and was analyzed by GraphPad Prism 7.0 software (San Diego, CA, United States). Student’s *t*-test was used to compare two groups, and multiple groups were analyzed by one-way ANOVA with Bonferroni tests. *p* < 0.05 was used to identify statistically significant.

Other experimental materials and methods were available in the supplementary materials.

## Results

### LH011 improved DSS-induced mice colitis

**FIGURE 1:**
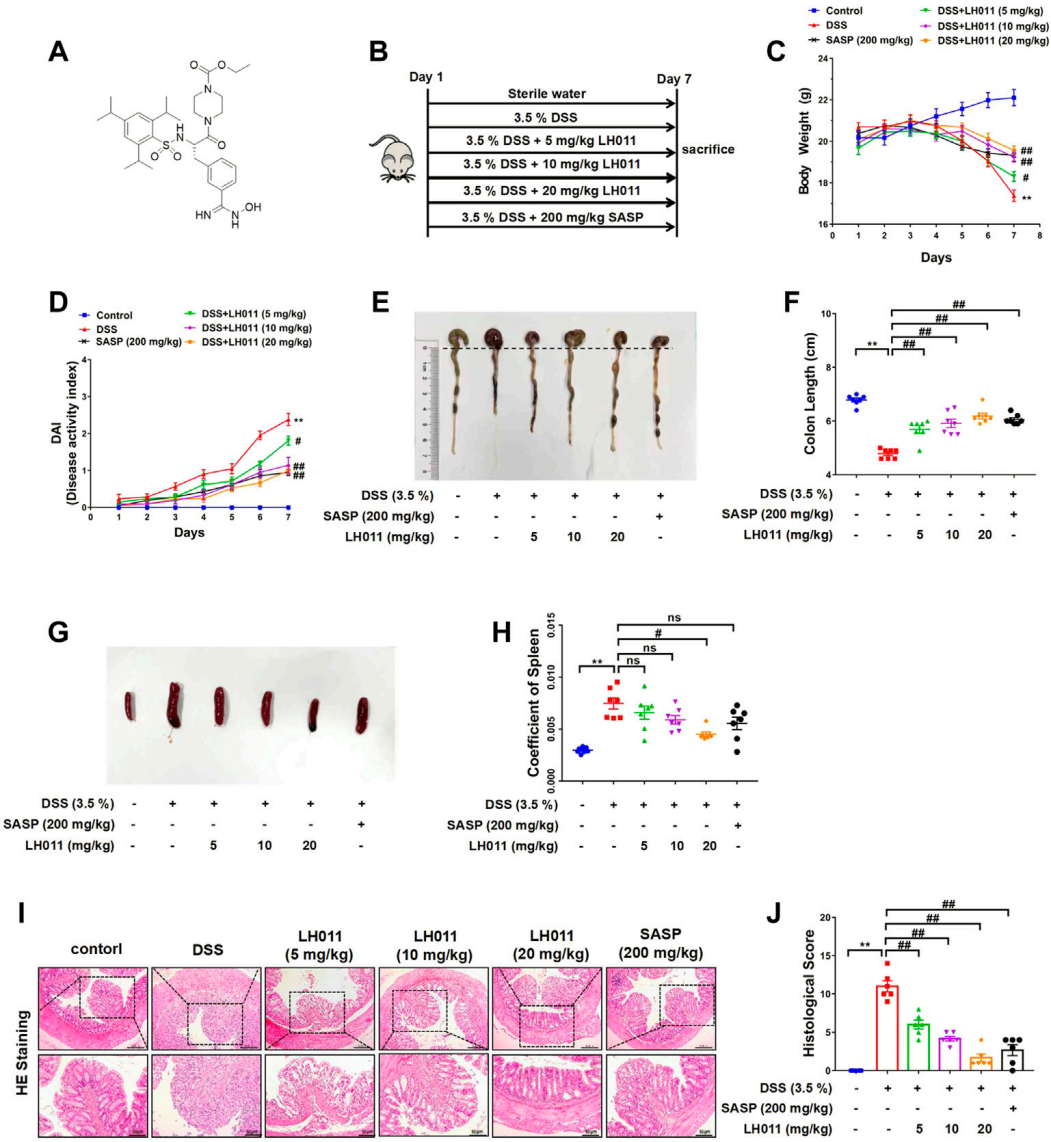
LH011 improved DSS-induced colitis in mice. **(A)** Chemical structure of LH011. **(B)** Diagram of animal experimental flow process. **(C)** Body weight changes. n = 9. **(D)** Disease activity index (DAI) scores n = 7. **(E)** Representative images of colons were shown. **(F)** Length of colons was measured and analyzed. n = 7. **(G)** Representative images of the spleen. **(H)** Coeffficent of the spleen in each group. n = 7. **(I)** Histological changes were detected by H&E staining, scale bar: 200 μm n = 6. **(J)** Histological scores of H&E in each group. n = 6. All data are presented as the mean ± SEM. ^**^ *p* < 0.01 vs. control group; ^#^ *p* < 0.05, ^##^ *p* < 0.01 vs. DSS group; ns, no significance.

LH011 was an inhibitor of trypsin and previously found acting as anti-inflammatory compound (Figure 1A). During UC, persistent inflammation and oxidative stress were the main features. Therefore, we explored the effects of LH011 on UC. DSS (3.5% w/v) was dissolved in the sterile water and freely drank by mice for 7 days to induce acute colitis. Meanwhile, LH011 was dissolved in saline with 1.5% absolute alcohol at different concentrations (5, 10, and 20 mg/kg) and administrated to mice by intraperitoneal injection. In addition, SASP (200 mg/kg, p. o.) was dissolved in 0.5% CMC-NA as the positive control. Mice in the control group were given sterile water and vehicle solvent (Figure 1B). Abnormal body weight loss, stool consistency, and bleeding are typical characteristics in the DSS-induced colitis (Yan et al., 2009). DAI is a comprehensive score including weight loss, stool consistency, and bleeding, which might indicate the severity of colitis. Therefore, we monitored the daily changes about body weight, stool consistency, and bleeding in mice. As shown in Figures 1C,D, DSS obviously decreased the body weight and increased the DAI scores of mice, which were significantly improved by LH011 in a dose-dependent manner. As shown in Supplementary Figure S1, the stool blooding of mice was severe as times went on in the DSS group, while that was obviously improved following LH011 or SASP treatment. Furthermore, DSS induced the shrink of the colon, while LH011 or SASP effectively maintained the length of the colon (Figures 1E,F). In addition, the spleen is the home of macrophage and the size of the spleen will be enlarged during inflammation (El-Shenawy et al., 2017). As shown in Figures 1G,H, the size of spleen was significantly increased following DSS stimulation, and LH011 or SASP effectively inhibited the enlargement of spleen. The damages of the colon could be further indicated by H&E staining, and our results showed that the DSS group mice showed serious falls off of the epithelium of intestinal mucosa, obvious lymphocyte infiltration of lamina propria, disappear or expand of the recess, and inflammatory infiltration. However, these histological damages were obviously improved in the LH011 or SASP treatment group (Figures 1I,J). All these results revealed the protective role of LH011 against DSS-induced colitis.

### LH011 relieved inflammatory response and oxidative stress in the DSS-induced mice colitis

Although the precise pathologic mechanisms mediating the progression of UC are still unclear, more and more evidence from previous studies indicated that excessive inflammation and oxidative stress were crucial to the pathogenesis of UC (Hilda et al., 2016). Subsequently, we measured the anti-inflammation and anti-oxidative stress effects of LH011.

**FIGURE 2:**
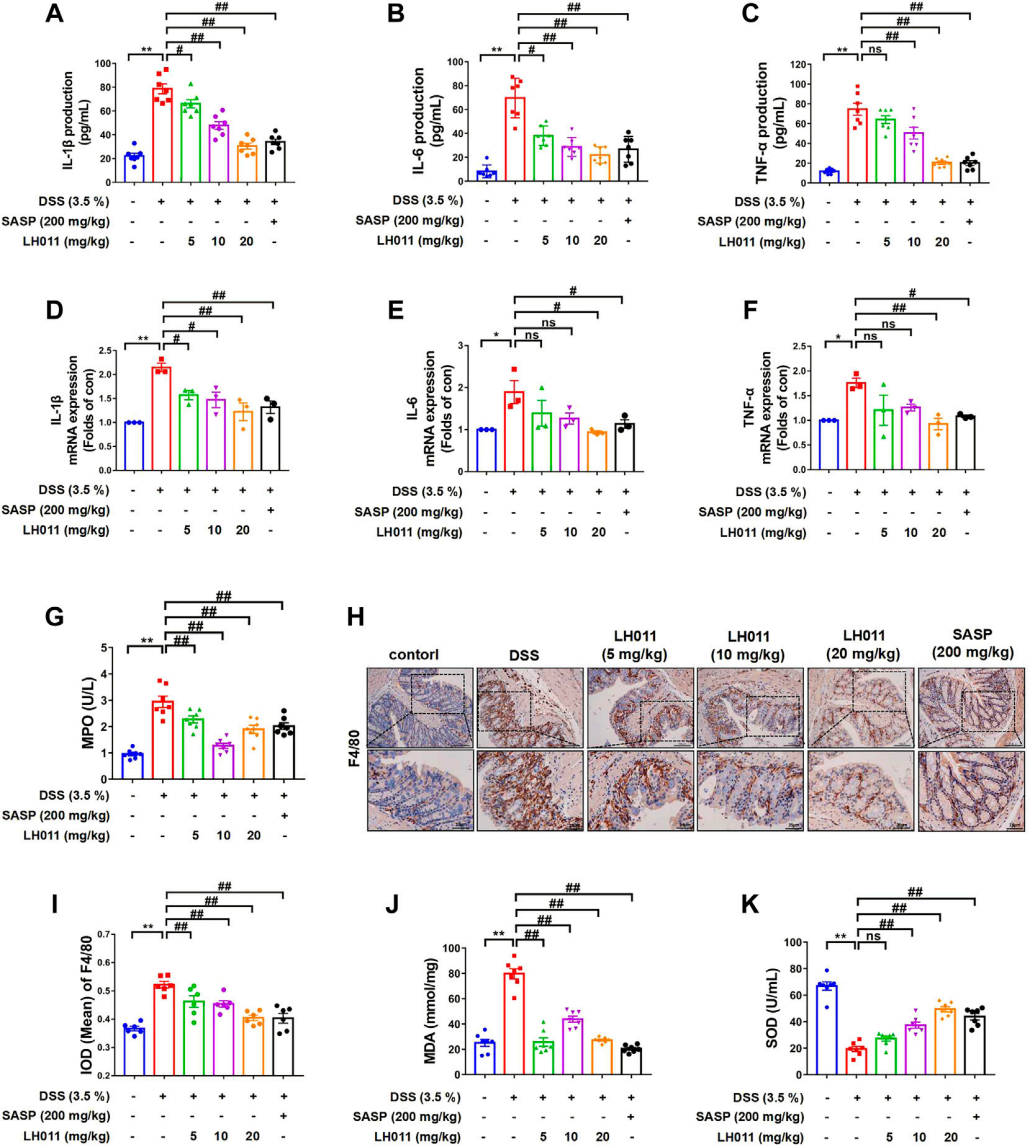
LH011 relieved inflammation and oxidative stresses in the colonic tissue. **(A–C)** Serum levels of IL-1β, IL-6, and TNF-α. n = 7. **(D–F)** mRNA levels of IL-1β, IL-6, and TNF-α in the colonic tissue. n = 3. **(G)** Levels of MPO in the colonic tissue. n = 7. **(H,I)** Representative IHC results of F4/80 to indicate the infiltration of macrophage, and the results were analyzed by Image Pro-Plus 6.0, scale bar: 100 μm n = 6. **(J,K)** Levels of MDA and SOD in the colonic tissues. n = 7. All data are presented as the mean ± SEM. ^*^ *p* < 0.05, ^**^ *p* < 0.01 vs. control group; ^#^ *p* < 0.05, ^##^ *p* < 0.01 vs. DSS group; ns, no significance.

It has been reported the production of extensive and massive inflammatory cytokines (e.g., IL-1β, IL-6, and TNF-α) are the characteristic of UC (Hamers et al., 2015). Alleviating inflammation *via* inhibiting expression of cytokines is an effective way in colitis mice (Xu et al., 2021). Our results showed that the serum levels of IL-1β, IL-6, and TNF-α were remarkably elevated following DSS stimulation (Figures 2A–C). However, LH011 or SASP could significantly relieve the elevation of those factors. LH011 or SASP also significantly reduced the mRNA levels of IL-1β, IL-6, and TNF-α in colonic tissues (Figures 2D–F). The degree of neutrophil infiltration could be determined by MPO activity index (Ferrat et al., 2018). As shown in Figure 2G, compared with the control group, MPO activity was significantly higher after DSS stimulation. Contrarily, the LH011 or SASP treatment significantly reduced the MPO activity. Furthermore, immunohistochemistry (IHC) results also showed that macrophages infiltration was increased in the DSS group as indicated by increased F4/80 staining positive results, which was also obviously relieved following LH011 or SASP treatment (Figures 2H,I).

It is well known that oxidative stress is also the main cause in the formation process of UC. MDA and SOD are significant indicators to evaluate the oxidative capacity. Here, we also measured the levels of MDA and SOD in colonic tissues. As shown in Figures 2J,K, LH011 and SASP improved DSS-induced elevation of MDA and reduction of SOD.

All these *in vivo* results indicated that LH011 could effectively relieve DSS-induced inflammation and oxidative stress.

### LH011 relieved inflammatory response and oxidative stress in LPS-induced RAW 264.7 cells

Activated RAW 264.7 cells participate in a variety of inflammatory responses. In the host’s defense system, inflammatory response plays a pivotal role. As an endotoxin, LPS can induce strong inflammatory signals, which help to increase the activation of monocytes/macrophages and the expressions of pro-inflammatory mediators including NO, PGE_2_, iNOS, COX2, and pro-inflammatory cytokines such as IL-1β, IL-6, and TNF-α (Choi et al., 2018).

**FIGURE 3:**
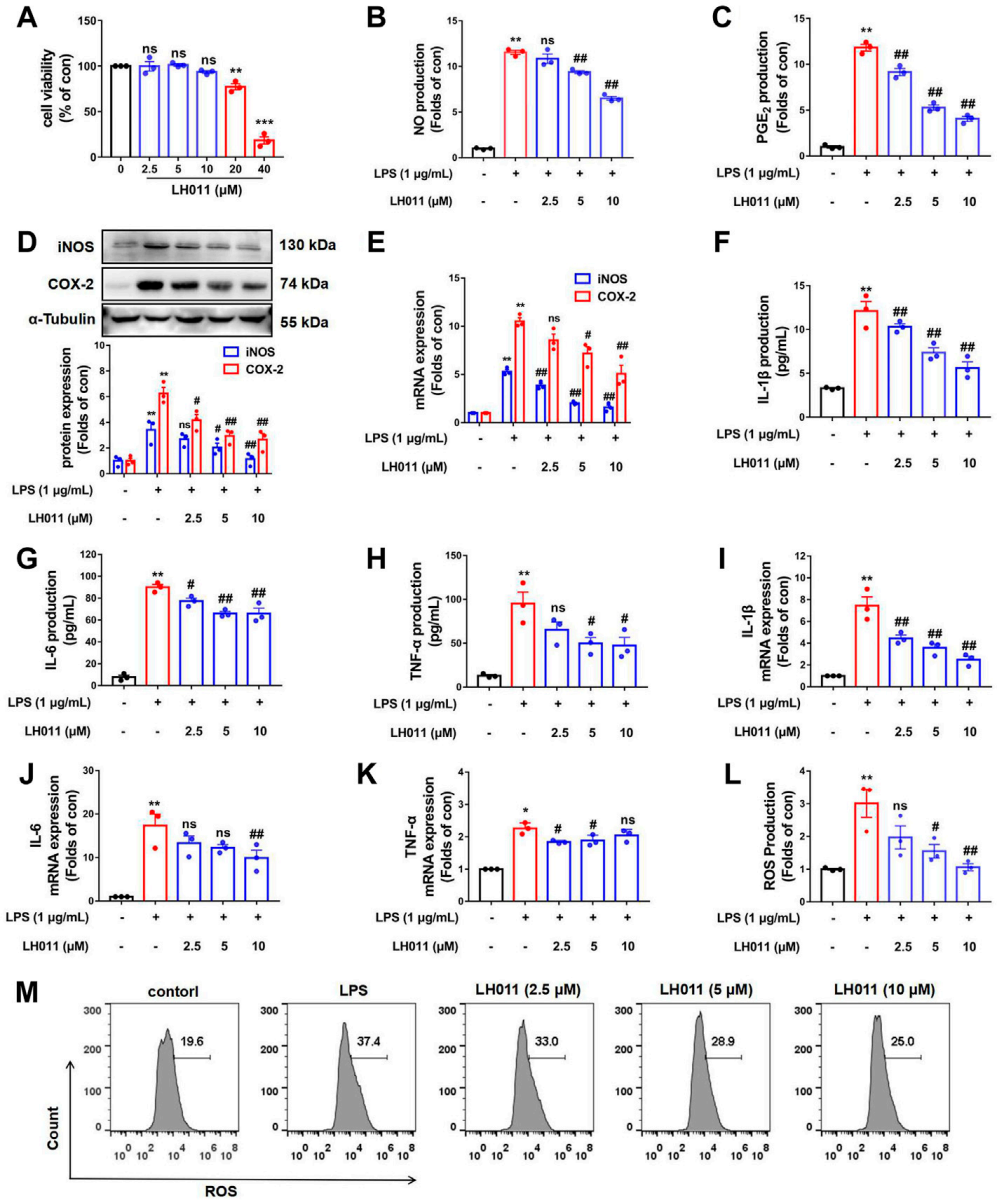
Effects of LH011 on the LPS-induced injury in RAW 264.7 cells. **(A)** RAW 264.7 cells were stimulated with different concentrations of LH011 for 24 h and cell viability was measured. **(B,C)** Concentrations of NO and PGE_2_ in the cultured medium were measured. **(D)** Protein levels of iNOS and COX-2. **(E)** mRNA levels of iNOS and COX-2. **(F–H)** Production of IL-1β, IL-6, and TNF-α. **(I–K)** mRNA levels of IL-1β, IL-6, and TNF-α. **(L,M)** Intracellular levels of ROS were detected by flow cytometry. All data are presented as the mean ± SEM (n = 3). ^*^ *p* < 0.05, ^**^ *p* < 0.01, ^***^ *p* < 0.001 vs. control group; ^#^ *p* < 0.05, ^##^ *p* < 0.01 vs. DSS group; ns, no significance.

To further validate the effects of LH011, LPS-induced RAW 264.7 cells *in vitro* were used to assay the effects of LH011 on anti-inflammation and anti-oxidative stress. First, the viability of RAW 264.7 cells was assayed with LH011 incubation at different concentrations. LH011 up to 10 μM did not influence the viability of cells (Figure 3A). Therefore, the appropriate concentrations of LH011 in the subsequent experiments were chosen at 2.5, 5, and 10 μM, respectively. To assess the anti-inflammatory effect of LH011, we further measured the changes of NO and PGE_2_ in LPS-induced RAW 264.7 cells. The results indicated that LH011 not only relieved LPS-induced secretion of NO in the cultured medium but also decreased the expression of PGE_2_ (Figures 3B,C). Moreover, it is well known that iNOS and COX-2 are positively related to the production of NO and PGE_2_. The more iNOS and COX-2 are expressed, the more inflammatory cytokines will be produced (Soufli et al., 2016). Our results showed that LH011 decreased the expressions of iNOS and COX-2 (Figures 3D,E). The ELISA and RT-qPCR were used to measure the release of pro-inflammatory cytokines. As shown in Figures 3F–H, LPS significantly induced the secretions of IL-1β, IL-6, and TNF-α. At the same time, the mRNA levels of IL-1β, IL-6, and TNF-α were augmented by LPS stimulation (Figures 3I–K). However, LH011 significantly relieved the expressions and secretion of IL-1β, IL-6, and TNF-α in a dose-dependent manner.

Generally, reactive oxygen species (ROS) is a natural by-product of normal metabolism and plays an important role in cell signaling transduction and appropriate physiological function. However, the production of large amounts of ROS is the characteristic of oxidative stress, and it can cause serious damage to cell structure, contribute to inflammatory response, and disease progression (Yun et al., 2018). To explain the role of LH011 in oxidative stress, we further measured the changes of ROS in LPS-induced RAW 264.7 cells. As shown in Figure 3L,M, compared with the control group, the flow cytometry results revealed that LH011 could effectively inhibit the increase of ROS induced by LPS in a dose-dependent manner.

These *in vitro* results validated that LH011 plays both anti-inflammatory and anti-oxidative roles.

### LH011 relieved inflammation by inhibiting TLR4/NF-κB pathway in DSS-induced mice colitis

**FIGURE 4:**
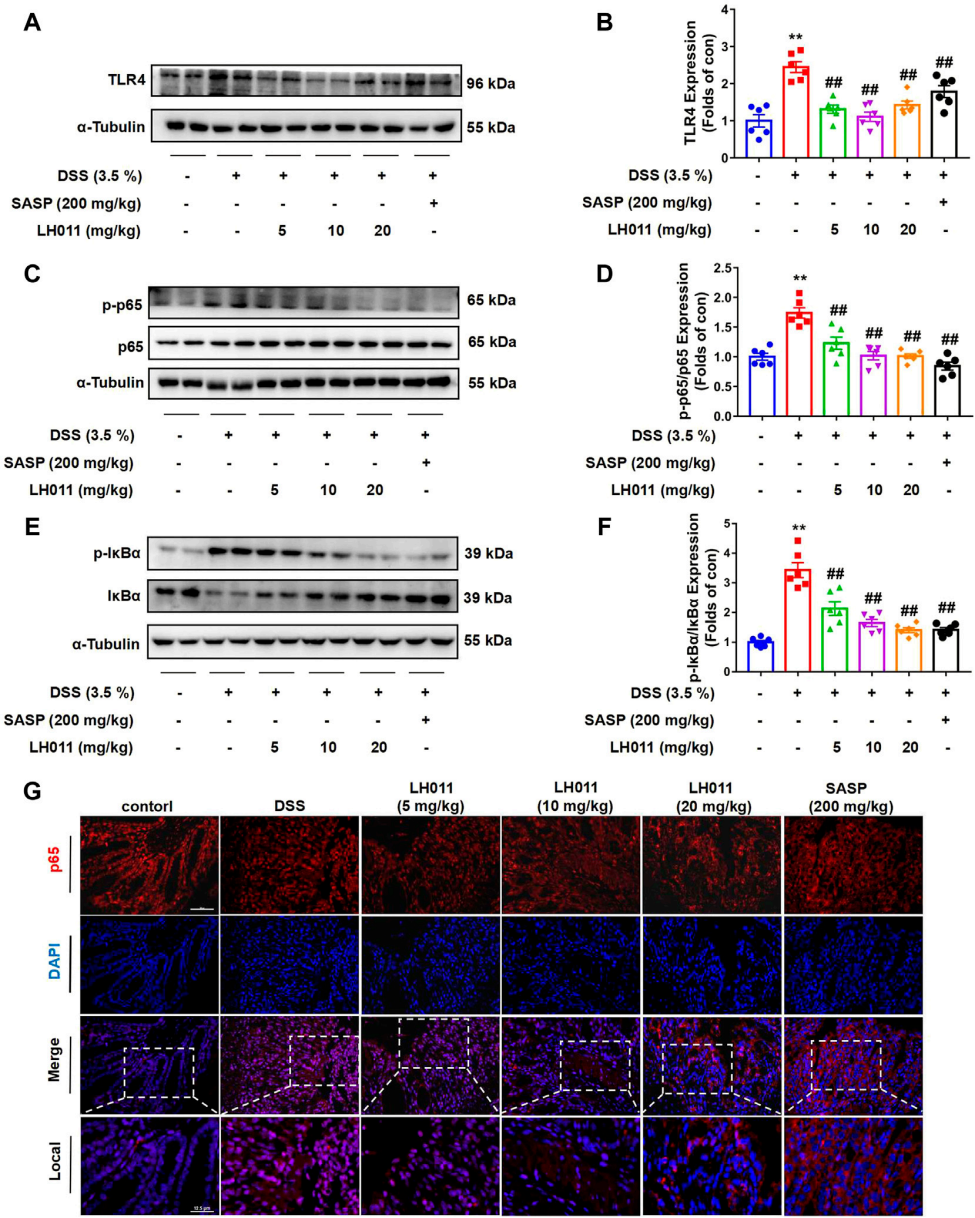
LH011 relieved inflammation by inhibiting TLR4/NF-κB signaling pathway in DSS-induced colitis mice. **(A–F)** Protein levels of TLR4, phosphorylated NF-κB p65, total p65, phosphorylated IκBα, and total IκBα in colonic tissue were measured by Western blot. n = 6. **(G)** Immunofluorescence results to indicate the sub-localization of NF-κB p65, scale bar: 50 μm, n = 3. All data are presented as the mean ± SEM. ^**^ *p* < 0.01 vs. control group; ^##^ *p* < 0.01 vs. DSS group.

Trypsin is closely related to inflammatory reaction (González-Titos et al., 2021). Since the 1960s, trypsin inhibitors have been used clinically to repair tissue for they can solve the inflammatory response (Shah and Mital, 2018). When trypsin binds to its corresponding receptor, it will activate many signaling pathways, including NF-κB, leading to the expression and secretion of a variety of inflammatory cytokines, such as IL-6 and TNF-a (Chen et al., 2011). Therefore, we speculated that LH011 exerts an anti-inflammatory effect through the NF-κB pathway. During inflammation, the TLR4/NF-κB signaling pathway is activated to induce the expressions of IL-1β, IL-6, and TNF-α (Li et al., 2021). The Western blots results showed that DSS increased the protein expression of TLR4 and phosphorylation of NF-κB p65 and IκBα while inhibited the total protein of IκBα. However, LH011 or SASP treatment not only markedly decreased DSS-induced protein expression of TLR4 but also decreased the phosphorylation of NF-κB p65 and IκBα (Figures 4A–F). IF staining of colonic tissue showed that DSS increased the nuclear localization of NF-κB p65. While treating with the LH011 or SASP, the nuclear localization of NF-κB p65 was markedly reversed (Figure 4G). These results indicated that LH011 could effectively inhibit the TLR4/NF-κB signaling pathway in DSS-induced mice colitis.

### LH011 inhibited LPS-induced TLR4/NF-κB pathway activation in RAW 264.7 cells

**FIGURE 5:**
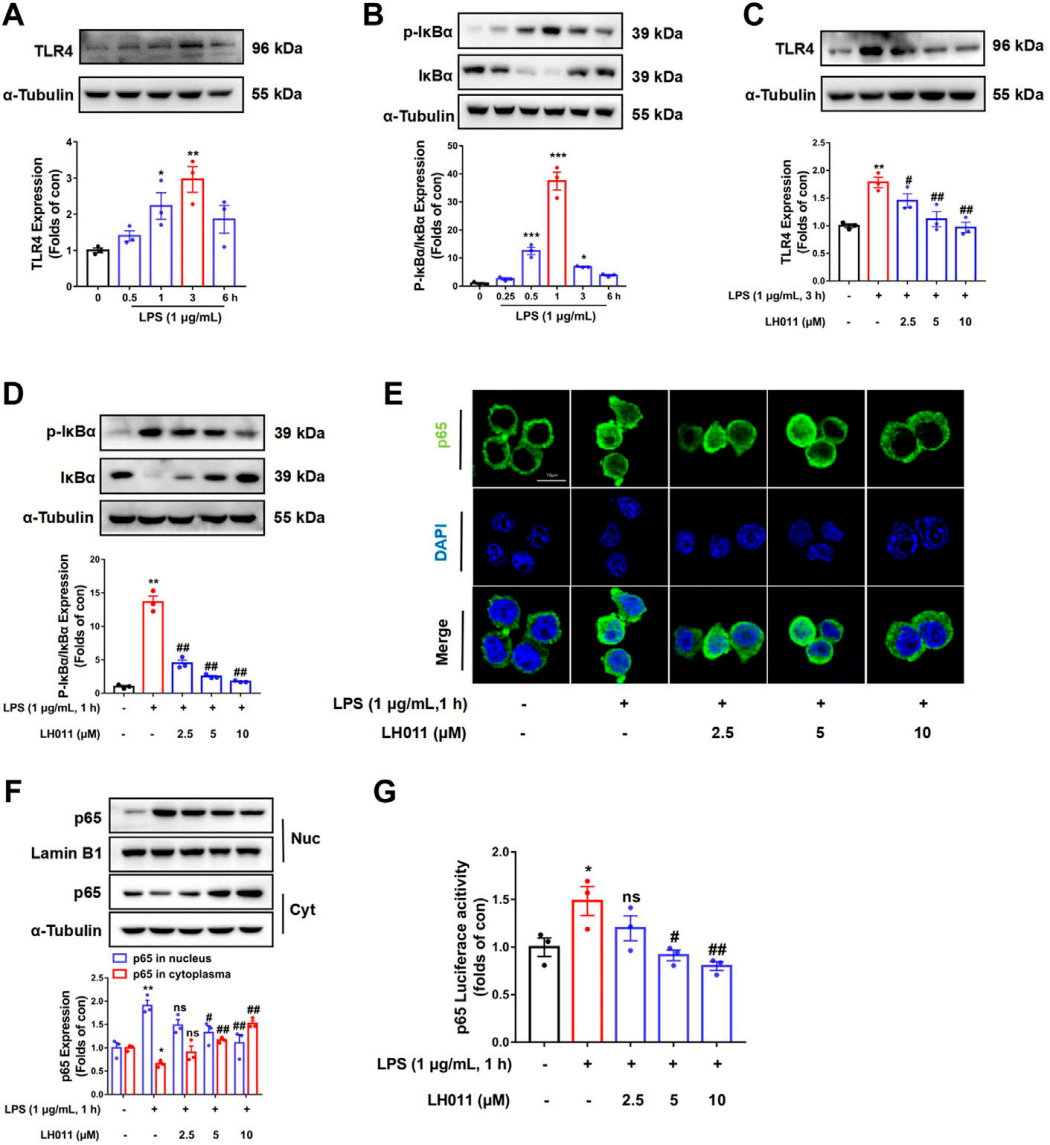
LH011 inhibited LPS-induced TLR4/NF-κB signal activation in RAW 264.7 cells. LPS stimulated RAW 264.7 cells at different time points. **(A,B)** Protein levels of TLR4, phosphorylated IκBα, and total IκBα were measured by Western blot. **(C)** Protein level of TLR4 was measured in RAW 264.7 cells with LPS stimulate at 3 h. **(D)** Protein level of p-IκBα and total IκBα were measured in RAW 264.7 cells, with LPS stimulate at 1 h with co-treatment with LH011. **(E)** Sub-localization of NF-κB p65 was measured by immunofluorescence staining with LPS stimulate at 1 h with co-treatment with LH011, scale bar: 10 μm. **(F)** Nucleic protein was extracted and the level of NF-κB p65 was measured with LPS stimulate at 1 h with LH011 co-treatment. **(G)** Transcriptional activity of NF-κB p65 was measured by dual-luciferase reporter assay with LPS stimulate at 1 h with LH011 co-treatment. All data are presented as the mean ± SEM (n = 3). ^*^ *p* < 0.05, ^**^ *p* < 0.01, ^***^ *p* < 0.001 vs. control group; ^#^ *p* < 0.05, ^##^ *p* < 0.01 vs. LPS group; ns, no significance.

It is well known that NF-κB is a classic signaling pathway and plays a key role in LPS-induced inflammation (Doyle and O'Neill, 2006). Therefore, we further monitor the effects of LH011 on the TLR4/NF-κB signaling pathway *in vitro*. Once the cell membrane receptor TLR4 is activated by LPS, IκBα will be phosphorylated by activated IκB kinase (IKK) and degraded *via* the subsequent ubiquitin-proteasome pathway (Karin et al., 2004). As shown in Figure 5A, LPS could increase the expression of TLR4, which reach peak at 3 h. The NF-κB pathway is a classic inflammatory signaling pathway downstream of TLR4. LPS-stimulation will dynamically induce the phosphorylated IκBα. As shown in Figure 5B, we found that the protein level of p-IκBα/IκBα increased with LPS-stimulation for 0.5–3 h and reached a peak at 1 h. Therefore, we speculated that the degradation period of TLR4 is significantly longer than the degradation of IκBα. Consequently, the protein peak level of TLR4 is inconsistent with p-IκBα/IκBα. In the next study, the expression of TLR4 and the phosphorylation of IκBα were, respectively, measured following LPS-stimulation at 3 and 1 h. As shown in Figures 5C,D, LH011 could relieve LPS-induced TLR4 expression and phosphorylation of IκBα in RAW 246.7 cells. The NF-κB p65 subunit could be released following IκBα phosphorylation and translocate into the nucleus (Guijarro-Muñoz et al., 2014). Next, we measured the sub-localization changes of NF-κB p65. Both Western blot and IF staining results showed that LPS increased the nucleus accumulation of NF-κB p65, while LH011 decreased the localization of NF-κB p65 in the nucleus (Figures 5E,F). Moreover, the dual-luciferase reporter assay result further confirmed that LH011 could inhibit the transcriptional activity of NF-κB p65 (Figure 5G).

### LH011 enhanced anti-oxidant defense by activating Nrf2/Keap1/HO-1 pathway in DSS-induced mice colitis

**FIGURE 6:**
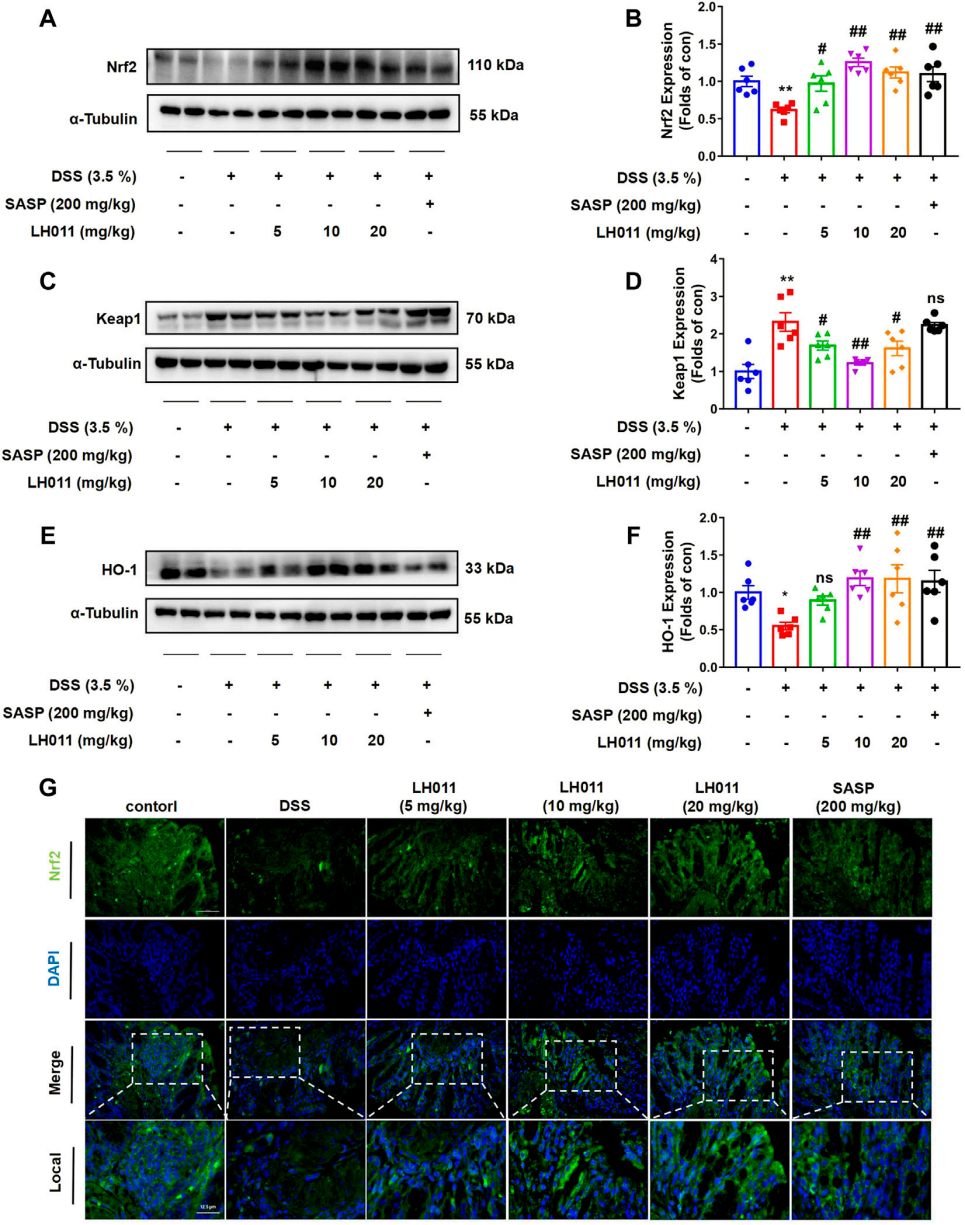
LH011 enhanced antioxidant defenses by activating Nrf2/Keap1/HO-1 signaling pathway in DSS-induced mice colitis. **(A–F)** Protein levels of Nrf2, Keap1, and HO-1 in colonic tissue were measured by Western blot. n = 6. **(G)** Immunofluorescence results of Nrf2, scale bar: 50 μm, n = 3. All data are presented as the mean ± SEM. ^*^ *p* < 0.05, ^**^ *p* < 0.01 vs. control group; ^#^ *p* < 0.05, ^##^ *p* < 0.01 vs. DSS group; ns, no significance.

Nrf2 plays a pivotal role in oxidative stress and regulates the transcription of anti-oxidative genes (Kobayashi et al., 2013). Nrf2 is constitutively degraded through binding to an adapter protein of E3 ubiquitin ligase Kelch-like ECH-associated protein 1 (Keap1). However, during the oxidative condition, Nrf2 will be released, escape from degradation, and results in a rapid nucleic accumulation. In the nucleus, Nrf2 forms a heterodimer with one of the small Maf proteins to promote the transcription of anti-oxidative genes, such as HO-1 (Gerstgrasser et al., 2017). Therefore, to explain the role of LH011 on oxidative stress, we measured the changes of the Nrf2/Keap1/HO-1 signaling pathway. As shown in Figures 6A–F, DSS significantly increased the protein level of Keap1 and inhibited the expressions of Nrf2 and the downstream target protein HO-1. However, LH011 or SASP could effectively decrease the protein level of Keap1 and further increase the protein levels of Nrf2 and HO-1 in DSS-induced colitis. Moreover, the effect of LH011 was stronger than that of SASP. In addition, IF staining results from colonic tissue further validated LH011, which increased the protein level of Nrf2 and its nucleic accumulation (Figure 6G). These results showed that LH011 relieved oxidative stress in colitis mice by activating the Nrf2/Keap1/HO-1 signaling pathway.

### LH011 increased Nrf2/Keap1/HO-1 pathway activation in LPS-induced RAW 264.7 cells

**FIGURE 7:**
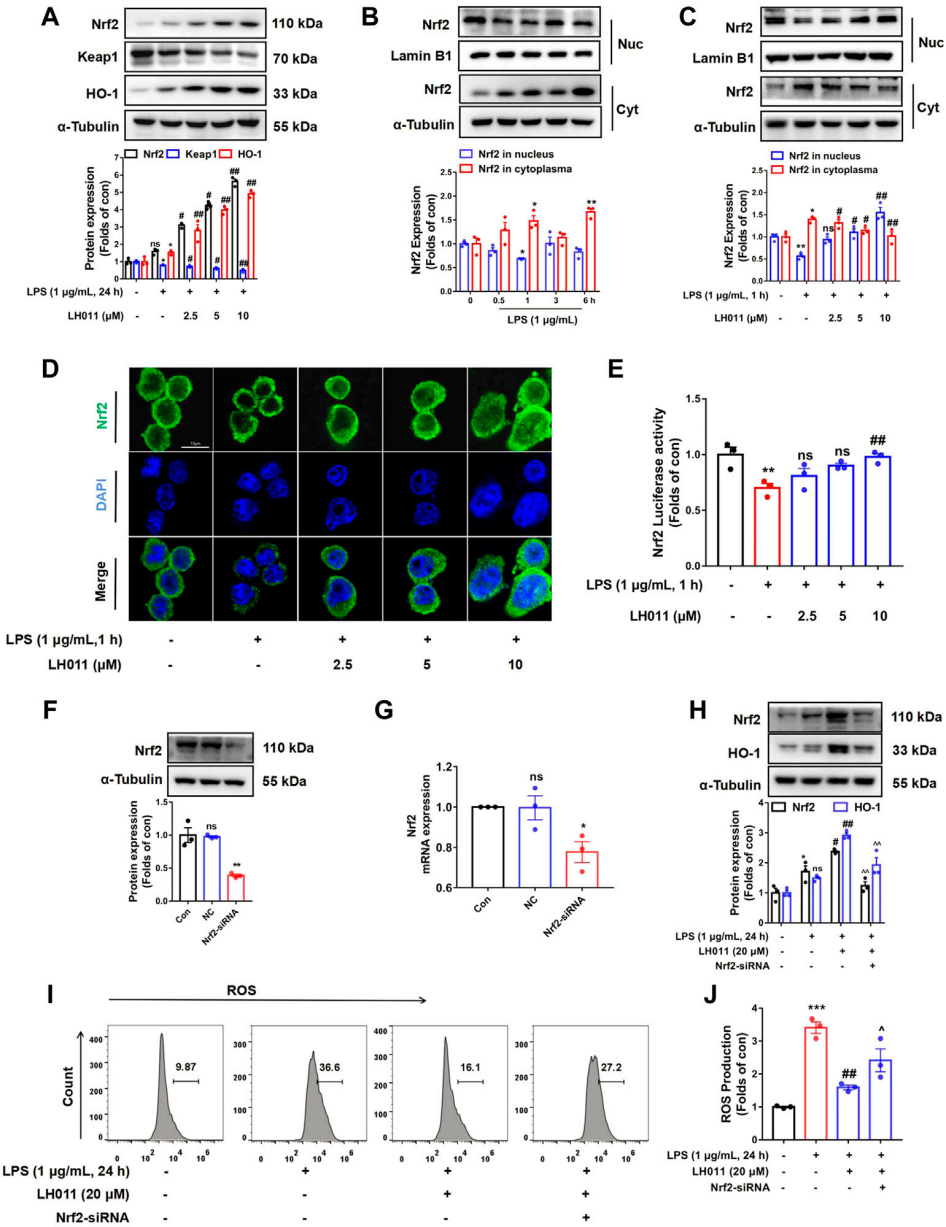
LH011 increased Nrf2/Keap1/HO-1 activation in RAW 264.7 cells. **(A)** Protein levels of Nrf2, Keap1, and HO-1 were measured by Western blot in RAW 264.7 cells. **(B)** Nucleic protein was extracted and the level of Nrf2 was measured with LPS stimulate at different time points. **(C)** Nucleic protein was extracted and the level of Nrf2 was measured with LPS and LH011 co-stimulate. **(D)** Sub-localization of Nrf2 was measured by immunofluorescence, scale bar: 10 μm. **(E)** Transcriptional activity of Nrf2 was measured by dual-luciferase reporter assay with LPS stimulate at 1 h. **(F,G)** Nrf2 was silenced by targeting siRNA. The protein and the mRNA levels of Nrf2 were measured. **(H)** Protein changes of Nrf2 and HO-1 were measured by Western blot. **(I,J)** Change of ROS was detected by flow cytometry. All data are presented as the mean ± SEM (n = 3). ^*^ *p* < 0.05, ^**^ *p* < 0.01, ^***^ *p* < 0.001 vs. control group; ^#^ *p* < 0.05, ^##^ *p* < 0.01 vs. LPS group. ^^^ *p* < 0.05, ^^^^ *p* < 0.001 vs. LPS with LH011; ns, no significance.

As a major transcription factor, Nrf2 protects cells from inflammation and oxidative stress caused by excessive ROS by activating downstream genes, including HO-1 (Loboda et al., 2016). As shown in Figure 7A, when stimulated with LPS for 24 h, the protein expressions of Nrf2 and HO-1 were increased, while the protein level of Keap1 was decreased in the LH011 group. However, LPS stimulation alone could slightly increase the expressions of Nrf2 and HO-1 and decrease the protein level of Keap1. Therefore, the increased expression of Nrf2 might be an adaptive response as the nuclear localization of Nrf2 was significantly decreased with LPS stimulation for 1 h (Figure 7B). Furthermore, Western blot and IF staining results exhibited that the localization of Nrf2 in the nucleus increased following LH011 administration (Figures 7C,D). In addition, the dual-luciferase reporter assay also confirmed that LH011 could augment the transcriptional activity of Nrf2 (Figure 7E).

To further explore whether Nrf2 mediated the protective effects of LH011, we treated RAW 264.7 cells with LH011 while Nrf2 knocked down. Western blot and qPCR results showed that Nrf2 was successfully knocked down by the targeting siRNA (Figures 7F,G). As shown in Figure 7H, silencing of Nrf2 inhibited the expression of HO-1 by LH011 in LPS-stimulated RAW 264.7 cells. Furthermore, the depletion of Nrf2 reversed the downregulation of ROS by LH011 in RAW 264.7 cells with LPS stimulation for 24 h (Figures 7I,J).

Our results illustrated that LH011 could significantly increase the activation of the Nrf2/Keap1/HO-1 signaling pathway in RAW 264.7 cells induced by LPS.

## Disscusion

**FIGURE 8:**
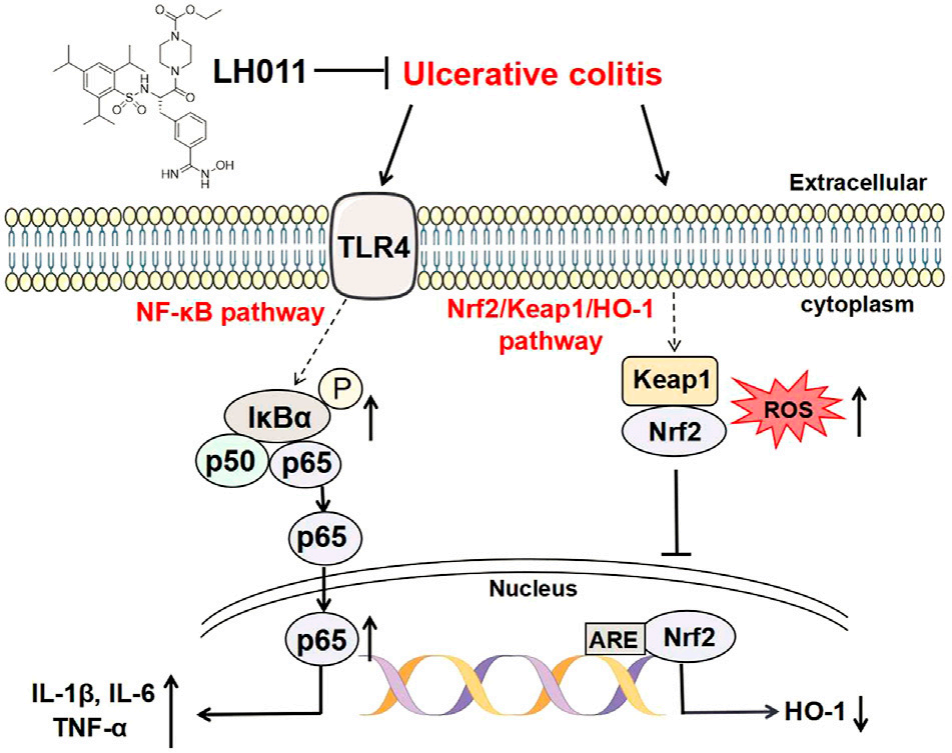
Schematic diagram of LH011 in treating UC. In conclusion, current experimental results suggested that LH011 could improve DSS-induced mice colitis. On the one hand, LH011 decreased inflammatory response by inhibiting the TLR/NF-κB signaling pathway. On the other hand, LH011 enhanced anti-oxidant capacity by activating the Nrf2/Keap1/HO-1 signaling pathway.

As shown in Figure 8, the current research showed that LH011 could effectively improve mice colitis. Mechanically, LH011 could alleviate inflammation and oxidative stress by inhibiting the TLR/NF-κB signaling pathway and activating the Nrf2/Keap1/HO-1 signaling pathway. LH011 may become a new compound for the treatment of ulcerative colitis.

DSS is an anionic polyionic derivative, which is composed of sulfated polysaccharides and could bind to colonic medium chain fatty acids to damage the intestine and lead to inflammation (Chassaing et al., 2014). Meanwhile, it has been reported that the UC model induced by 2–5% DSS is similar to human UC and easy to replicate (Eichele and Kharbanda, 2017). To determine the appropriate concentration of DSS, 2, 3.5, and 5% DSS were used for modeling in the pre-experiment. It was found that the modeling of the 2.5% DSS group was not obvious, and mice in the 5% DSS group were in a poor state. However, mice in the 3.5% DSS group were in a normal state, and the modeling of 3.5% DSS was replicated successfully. In this study, 3.5% DSS (in the sterile water) successfully induced mice colitis as indicated by remarkably higher DAI score and typical pathological changes in the colonic tissue.

Macrophages participated in non-specific defense (innate immunity) and specific defense (cellular immunity) in vertebrates, and it plays an important role in the early development and defense of intestinal inflammation (Muller et al., 2020). Macrophages not only exhibit diverse functions, but also have high plasticity. Macrophages mainly are divided into M1 and M2 types, according to different activation states and functions (Geissmann et al., 2010). M1 type macrophage, a classically activated macrophage, is induced by activated irritants, such as IFN-γ or LPS. The activated M1 type macrophages exhibit the ability to promote inflammation. As the anti-inflammatory type, M2 macrophage is stimulated by IL-4, IL-10, and IL-13 (Wynn and Barron, 2010). In our results, inflammatory response and macrophage infiltration in colonic tissue of the DSS group were significantly increased, but significantly relieved following LH011 treatment. Raw 264.7 cell is one of the most commonly used *in vitro* models for inflammation research. LPS is a substance of the cell wall of Gram-negative bacteria, which is composed of lipids and polysaccharides. As it can trigger the cascade reaction in the process of inflammation, LPS is usually used to experimentally induce macrophage activation (Putker et al., 2015). In this study, we established the *in vitro* model by stimulating RAW 264.7 cells with LPS to evaluate the effects of LH011.

Sulfasalazine (SASP) is a colon-specific pro-drug commonly used for the treatment of UC. SASP was orally absorbed and splitted into the therapeutic moiety 5-aminosalicylic acid (5-ASA), therefore inhibiting the expression of cytokines and NF-κB signaling pathway (Jo et al., 2005). In this study, SASP was used as positive drug and significantly attenuated DSS-induced colitis.

Phase Ⅱ clinical trial of LH011 has been conducted in pancreatic cancer and breast cancer. However, as a trypsin inhibitor, LH011 may more be proper to be developed into an anti-inflammatory agent. Our previous studies have shown the anti-inflammatory effect of LH011 on acute lung injury and acute pancreatitis (data were not shown). UC was a typical disease with persistent inflammation and oxidative stress in colonic tissue (Hilda et al., 2016). Therefore, the anti-inflammatory effects of LH011 were further evaluated in the UC. In this study, LH011 inhibited inflammatory reaction *in vivo* and *in vitro.* Chronic toxicity studies were carried out in rats for 4 and 26 weeks to evaluate the toxicity of LH011. The no observed effect level (of toxin) at 4 and 26 weeks was 30 mg/kg. Moreover, LH011 improved DSS-induced colitis in mice when the dosage was 10 mg/kg in the pre-experiment. Therefore, in this study, the concentrations of LH011 were 5, 10, and 20 mg/kg *in vitro.*

Trypsin prefers to activate protease-activated receptor 2 (PAR2) (González-Titos et al., 2021). Activated PAR2 regulates many signaling pathways involving in the pathogenesis of inflammatory disorders, such as the NF-κB pathway (Banfi et al., 2009). When LPS binds to TLR4 in the macrophage membrane, the NF-κB pathway will be activated and plays a key role in inflammatory processes (Kim et al., 2005). NF-κB is usually inactivated by binding to the inhibitory protein IκBα in cytoplasm. When upstream signal factors combine with cell membrane surface receptors, IκBα is phosphorylated and degraded, and then activated NF-κB p65 exposes the nuclear localization sequence (NLS), rapidly enters the nucleus from the cytoplasm and promotes the transcription of related inflammatory genes (Kawai and Akira, 2010). Many studies have shown that it is an effective means to alleviate UC by inhibiting the NF-κB signaling pathway (Hou et al., 2020). Our data revealed that LH011 inhibited the expression of TLR4, phosphorylation of IκBα, and the translocation to the nucleus of NF-κB p65. All these results showed that LH011 could play a significant role in inflammatory responses *via* suppressing the activation of the TLR4/NF-κB pathway.

Furthermore, the antioxidant role of LH011 was discovered. In this study, LH011 effectively relieved oxidative stress both *in vivo* and *in vitro*. Notably, Nrf2 and NF-κB are closely related. In the previous studies, Nrf2 played a significant role in lightening the inflammation, which was associated with inhibiting the NF-κB signaling pathway (Sivandzade et al., 2019). Generally, Nrf2 always bound with Keap1 in the cytoplasm and will be degraded by the ubiquitin proteasome pathway. Once released, Nrf2 will transfer into the nucleus and activate the transcription of the target genes (e.g., HO-1) (Jaiswal, 2004). In this study, LH011 effectively augmented the role of Nrf2 as indicated by the increased nucleic accumulation and transcriptional activity of Nrf2. Furthermore, the anti-oxidative capacity of LH011 was reversed with Nrf2 knockdown. Our data firmly showed that LH011 could effectively activate the Nrf2 signal pathway to protect against oxidative stress during UC.

## Data Availability

The original contributions presented in the study are included in the article/Supplementary Materials; further inquiries can be directed to the corresponding authors.

## Glossary

ARE: antioxidant response element
5-ASA: 5-aminosalicylic acid
BSA: bull serum albumin
COX-2: cyclo-oxygen-ase-2
CMC-NA: carboxymethyl cellulose
DAI: disease activity index
DSS: dextran sulfate sodium
ECL: enhanced chemiluminescence
ELISA: enzyme-linked immunosorbent assay
HO-1: heme oxygenase 1
HE: hematoxylin–eosin
HRP: horse radish peroxidase
IFN-γ: interferon-γ
IL-1β: interleukin-1β
IF: immunofluorescence
IBD: inflammatory bowel disease
IHC: immunohistochemistry
LPS: lipopolysaccharide
MyD88: myeloid differentiation response 88
MDA: malondialdehyde
Nrf2: NF-E2-related factor-2
NFκB: nuclear factor κB
NO: nitric oxide
PGE2: prostaglandin E2
PAR2: protease-activated receptor 2
ROS: reactive oxygen species
SASP: sulfasalazine
SDS–PAGE: sodium dodecyl sulfate polyacrylamide gel electrophoresis
SOD: superoxide dismutase
uPA: urokinase type plasminogen activator
TLR: toll-like receptor
TNF-α: tumornecrosisfactor-α
UC: ulcerative colitis.
