# Supplementary material for: Trypsin inhibitor LH011 inhibited DSS-induced mice colitis via alleviating inflammation and oxidative stress
Source: Front Pharmacol. 2022 Sep 27;13:986510. doi: 10.3389/fphar.2022.986510 (PMC9551103; doi:10.3389/fphar.2022.986510)
Supplement: Supplementary file 1 [file DataSheet1.docx]

Supplementary Material

**Trypsin inhibitor LH011 inhibited DSS-induced mice colitis via alleviating inflammation and oxidative stress**

^1^ Laboratory of Pharmacology & Toxicology, School of Pharmaceutical Sciences, Sun Yat-sen University, Guangzhou 510006, China

^2^ School of Pharmaceutical Sciences, Guangzhou Medical University, Guangzhou511436, China

^3^ Department of National and Local United Engineering Lab of Druggability and New Drugs Evaluation, School of Pharmaceutical Sciences, Sun Yat-Sen University, Guangdong 510006, China

^4^ Guangdong Provincial Key Laboratory of New Drug Design and Evaluation, School of Pharmaceutical Sciences, Sun Yat-Sen University, Guangdong, China

^5^ Guangdong Provincial Key Laboratory of Chiral Molecule and Drug Discovery

^6^ Guangzhou Link Health Group, Guangdong, China

***Corresponding author**

**Email address**: [chenjwen@mail.sysu.edu.cn](mailto:chenjwen@mail.sysu.edu.cn) (Jianwen Chen) & [liupq@mail.sysu.edu.cn](mailto:liupq@mail.sysu.edu.cn) (Peiqing Liu) & [y.song@healthinlink.com](mailto:y.song@healthinlink.com) (Yan Song)

**^#^ These authors contributed equally to this study**

**1 SUPPLEMENTARY MATERIAALS AND METHODS**

- 1. **Stool bleeding assay**

The stool of mice was smeared on the test card. Chromogenic agent A was dropped on the test card, and then Chromogenic agent B was dropped after agent A was completely penetrated. Observed and recorded the results according to the manufacturer’s instructions.

**1.2 Cell viability**

Cell viability was determined by CCK-8 kit (Beyotime, Shanghai, China). Briefly, Raw 264.7 cells were placed in 96-well plates and incubated overnight at 37 ℃ with 5% CO_2_ for 24 h. Then incubated with different concentrations of LH011 for 24 h. CCK8 reagent (10 %) was added to each well and incubated with cell at 37 ℃ for 1 h. The absorbance was read at 570 nm on a microplate reader (Epoch, BioTek, USA).

**1.3 Reactive oxygen species (ROS) assay**

To detect the ROS level in RAW 264.7 cells, we used a commercial ROS assay kit (Beyotime, Shanghai, China). Treated RAW 264.7 cells were incubated with DCFH-DA (10 μM) in the dark in 37 ℃ for 30 min according to the manufacturer’s instructions and then detected by flow cytometry.

**1.4 Cytokines analysis by enzyme-linked immunosorbent assay (ELISA)**

Serum from mice for each group were used to perform ELISA. The levels of TNFα, IL-6, and IL-1β were assayed by commercial ELISA kits (MultiSciences, Hangzhou, China). RAW 264.7 cells (1×10^4^ cells/mL) were incubated with LH011 at different concentrations (2.5, 5, 10 μM) for 24 h and then co-treated with LPS (1 μg/mL) for the last 1 h. TNF-α, IL-6, and IL-1β levels in the culture medium were determined using ELISA kits (MultiSciences, Hangzhou, China) according to the manufacturer's instructions.

**1.5 Immunofluorescence (IF) staining**

RAW 264.7 cells (1×10^4^ cells/mL) were seeded on coverslips in 12-well plates and cultured overnight. Cells were fixed with 4% paraformaldehyde for 15 min, incubated with 0.3% Triton X-100 (BioFroxx, Einhausen, Germany) for 10 min and then blocked with goat serum for 1 h. Primary antibodies for NF-κB p65 (1:200) and Nrf2 (1:200) were incubated with cell overnight at 4 ℃ overnight. Subsequently, second-fluorescence antibody was incubated with cell for 1 h at room temperature. Cell imaging was captured by confocal laser scanning microscope (Olympus Corp., Tokyo, Japan).

**1.6 Dual Luciferase reporter assay**

The DNA transcriptional activity of NF-κB p65 and Nrf2 were determined by the Dual Luciferase reporter assay (Beyotime, Shanghai, China) according to the manufacturer’s instructions. Briefly, Raw 264.7 cells were seeded in 96 well plates and then co-transfected with 0.2 μg pNF-κB-Luc or 0.2 μg pARE-luc (Beyotime, Haimen, China) and 0.02 μg pRL-TK (Promega, Madison, WI, USA) for 48 h, and then the cells were collected, the Luciferase activity was measured by the Dual Luciferase reporter assay system kit. The Luciferase activity was normalized to the Renilla Luciferase activity.

**2 SUPPLEMENTARY TABLES AND FIGURES**

**2.1 The changes of stool bleeding**

The stool blooding of mice was severe as times went on in the DSS group, which was obviously improved following LH011 or SASP treatment (Fig. S1).

**Fig. S1 (A) Representative images of stool blooding. n = 3.**


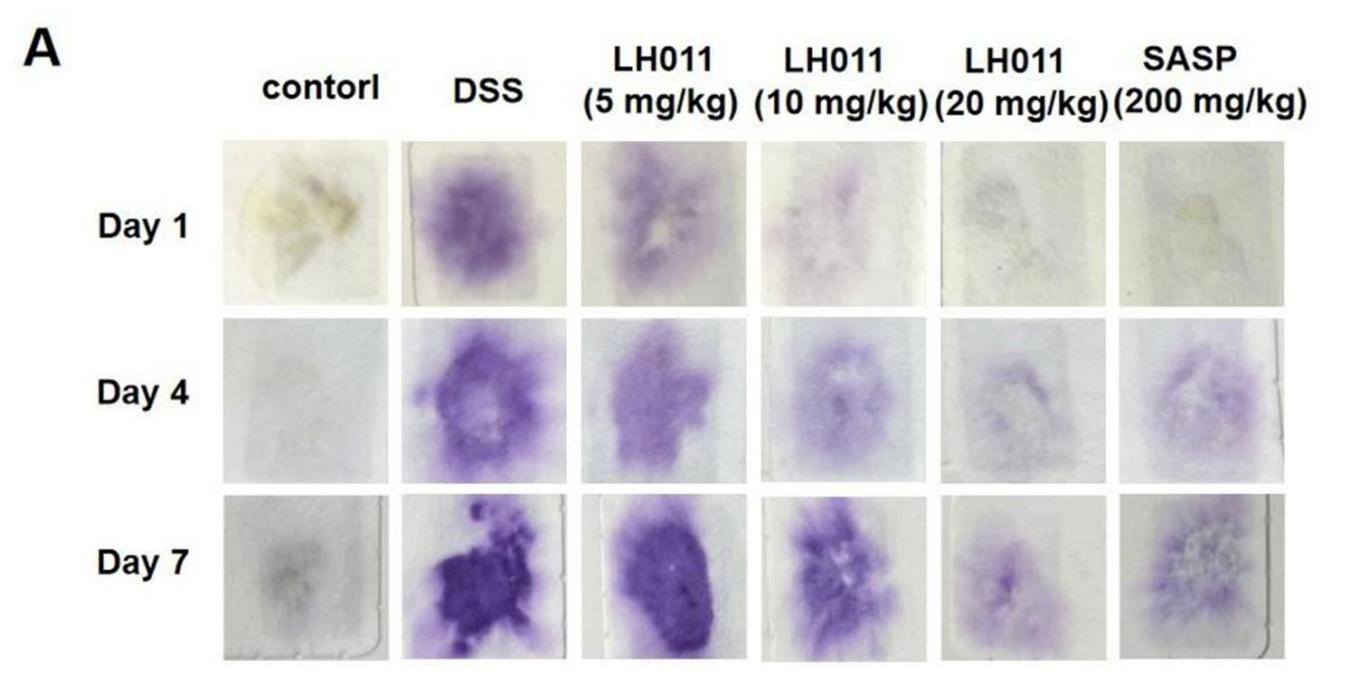


**2.2 Supplementary Table 1**

**The specific primers of sequences for qRT-PCR**

| **Gene** | **Forward sequence** | **Reverse sequence** |
| --- | --- | --- |
| IL-1β | TGCCACCTTTTGACAGTGATG | TGATGTGCTGCTGCGAGATT |
| IL-6 | TGATGGATGCTACCAAACTGGA | TGTGACTCCAGCTTATCTCTTGG |
| TNF-α | CCCTTTACTCTGACCCCTTTATTGT | TGTCCCAGCATCTTGTGTTTCT |
| iNOS | GGAGTGACGGCAAACATGACT | TCGATGCACAACTGGGTGAAC |
| COX-2 | GAAAGCCCTCTACAGTGACATC | GGTGCTCCAAGCTCTACCAT |
| β-actin | GTGAGGATGAGGAGAGCTATGA | CCTTTGGCCACTTTCCTCTTAT |
